# Umbilical Cord Blood Adiponectin, Leptin, Insulin, and Ghrelin in Premature Infants and Their Association With Birth Outcomes

**DOI:** 10.3389/fendo.2021.738964

**Published:** 2021-09-30

**Authors:** Luyan Han, Bo Li, Xiaojing Xu, Shufang Liu, Zhenghong Li, Ming Li, Danhua Wang

**Affiliations:** ^1^ Department of Pediatrics, The First Hospital of Tsinghua University, Beijing, China; ^2^ Department of Pediatrics, Peking Union Medical College Hospital, Peking Union Medical College and Chinese Academy of Medical Sciences, Beijing, China; ^3^ Department of Endocrinology, NHC Key Laboratory of Endocrinology, Peking Union Medical College Hospital, Peking Union Medical College and Chinese Academy of Medical Sciences, Beijing, China

**Keywords:** adipokine, insulin, umbilical cord blood, premature infants, neonatal growth

## Abstract

**Background:**

Premature/low-birth-weight infants are at significant risk of metabolic diseases in adulthood, which may be related to the levels of fetal adipokine. Here, we investigated the differences in the levels of umbilical cord blood adiponectin, leptin, insulin, and ghrelin in preterm and term infants and sought to elucidate the link between these hormones and fetal growth. We also evaluated the interrelationship among these metabolic hormones in both groups of newborns.

**Methods:**

A total of 149 mother–infant pairs (100 in the preterm group and 49 in the term group) were enrolled in the study. The preterm group was further subdivided according to birth weight (≤1,500, 1,501–2,000, 2,001–2,500, and >2,500 g), gestational age (<34 *vs.* ≥34 weeks), and appropriate for gestational age (AGA) *vs.* small for gestational age (SGA). The general condition of the mothers and the growth parameters of the newborns at birth were recorded.

**Results:**

The levels of adiponectin, leptin, and ghrelin were lower in the preterm group than those in the term group (*p* < 0.05). In the preterm group, the leptin levels of infants with gestational age ≥34 weeks were significantly higher than those of infants with gestational age <34 weeks (mean ln leptin = 0.63 *vs.* 0.36 ng/ml, *p* = 0.009). The levels of adiponectin were lower in the SGA group than those in the AGA group (mean ln adiponectin = 2.26 *vs.* 2.84 µg/ml, *p* = 0.001), whereas those of ghrelin displayed the opposite trend (mean ln ghrelin = 6.29 *vs.* 5.71 pg/ml, *p* < 0.001). Leptin was significantly correlated with insulin both in preterm infants with birth weight (BW) >2,000 g and in term infants. Umbilical cord blood leptin was positively correlated with the BW, birth length, and head circumference of newborns (*r* = 0.460, 0.311, and 0.310, respectively, all *p* < 0.05), whereas ghrelin was negatively correlated with the same parameters (*r* = −0.372, −0.415, and −0.373, respectively, all *p* > 0.05).

**Conclusions:**

The lack of maturation of adipose tissue and the gastrointestinal tract by the fetus due to prematurity is associated with changes in the levels of cord blood adiponectin, leptin, and ghrelin. The dysregulation of these hormones in preterm infants may be a risk factor for fetal growth and future metabolic diseases.

## Introduction

Premature infants are at high risk of developing metabolic syndrome-related disorders such as obesity, type 2 diabetes, and cardiovascular diseases in later life (1, 2); however, the mechanisms underlying the link between fetal growth and these metabolic disorders are not well understood. Investigating adipose tissue dysfunction may provide valuable insights into this association. Fetal adipose tissue maturation occurs in the second trimester of gestation, while its accumulation begins during the third trimester (3). As a consequence, prematurity can affect the correct acquisition of adipose tissue, thereby disturbing its metabolic functions and limiting the metabolic adaptation of premature newborns to extrauterine life. The adipokine profile is partially dependent on the amount of adipose tissue accumulated during fetal life, rendering cord blood adipokine levels potential markers of the degree of development and amount of adipose tissue.

Among the numerous adipokines derived from adipose tissue, adiponectin and leptin comprise a crucial signaling link between adiposity and metabolic disorders. Leptin has critical roles in the regulation of food intake and energy expenditure in white adipose tissue (4). Studies on cord blood have demonstrated that the levels of fetal leptin increase with advancing gestation (5, 6). Initially, the levels of fetal leptin are dependent on both transplacental delivery and placental leptin production until approximately 32 weeks of gestation. Subsequently, as it starts to accrue significant amounts of adipose tissue, the fetus becomes the main contributor to its own levels of plasma leptin (7). Adiponectin is an anti-inflammatory adipokine that regulates glucose metabolism and fatty acid oxidation (8). Circulating adiponectin exists in three forms, namely, trimers, hexamers, and high-molecular-weight oligomers, the latter being the most active and a relatively important isoform for promoting insulin sensitivity (9). Several studies have demonstrated that a negative correlation exists between maternal serum adiponectin levels and birth weight (BW) (10, 11).

In addition to the adipokines secreted by adipose tissue, insulin, produced in pancreatic islets, and ghrelin, produced primarily in the gastrointestinal mucosa, also play roles in fetal growth (12). The cross-talk between these hormones may be a contributing factor to fetal development (13, 14). Ghrelin stimulates appetite by binding to its specific receptor, GHS-1A, in the hypothalamus and activating NPY/AgRP-expressing neurons; it also participates in a meal-to-meal control system that is itself sensitive to changes in the levels of insulin and leptin (10). The influence of ghrelin on energy homeostasis raises the possibility that it may also play a role in prenatal growth.

Although numerous studies have explored the adiponectin and leptin levels in the umbilical cord blood of full-term infants (15–17), little is known about their homeostasis in preterm infants. Here, we focused on determining the differences in the levels of umbilical cord blood adiponectin, leptin, insulin, and ghrelin in preterm and term infants and sought to identify their possible influencing factors. Additionally, we investigated the interrelation among these metabolic hormones in both preterm and term infants and examined the relationship between these hormones and the neonatal growth parameters, thereby providing a possible theoretical basis for explaining how premature infants are prone to metabolic syndrome in later life.

## Materials and methods

### Study Population

Venous cord blood samples were collected from 149 newborns in the Department of Pediatrics and Obstetrics of Peking Union Medical College Hospital between August 2011 and December 2011. The mothers were 16 years of age or older, pregnant with a single fetus, and with no self-reported smoking and drinking history and diabetes, asthma, cancer, or psychiatric illness. According to gestational age (GA), the mother–infant pairs were divided into a preterm group (<37 weeks gestation) or a term group (37–41 weeks gestation). Preterm infants were also divided into different subgroups according to GA (<34 *vs.* ≥34 weeks) since several organ systems reach adequate maturity after 34 weeks (18). We also divided preterm infants according to BW (≤1,500, 1,501–2,000, 2,001–2,500, and >2,500 g), or small for gestational age (SGA) *vs.* appropriate for gestational age (AGA). The study procedures were approved by the Ethics Committee of Peking Union Medical College Hospital, and signed informed consent was obtained from all the participating families.

### Assessment of Maternal and Neonatal Characteristics

Measurements of infant BW, birth length, and head circumference were obtained immediately after birth. The GA was confirmed by ultrasound before week 20 of gestation. Maternal age, education, and gravidity were self-reported at the first research-related visit. Infant sex was reported by the mother shortly after delivery or deduced from the medical records. Neonatal umbilical cord blood was collected at delivery. The samples were centrifuged, aliquoted, and immediately frozen at −80°C until analysis of hormone levels. The levels of total ghrelin were tested using the total Human Ghrelin enzyme-linked immunosorbent assay (ELISA) kit (Millipore, Burlington, MA, USA) (19). The intra- and inter-assay coefficients of variation (CVs) for the ghrelin assay were <1.9% and 7.7%, respectively. The concentrations of adiponectin (20), leptin (21), and insulin (22) were measured using monoclonal antibody-based sandwich ELISA developed in the Key Laboratory of Endocrinology, Peking Union Medical College Hospital (Beijing, China). The intra- and inter-assay CVs were respectively <5.4% and <8.5% for adiponectin and <7.4% and <9.3% for leptin. The insulin assay showed no cross-reactivity to proinsulin (<0.05%), indicating that it was true insulin. The sensitivity of the insulin assay was 0.5 mU/L and the intra- and inter-assay CVs were <4.1% and <7.0%, respectively.

### Statistical Analysis

All analyses were performed using SPSS version 20.0 (IBM SPSS Statistics, Armonk, NY, USA). Data displaying skewed distributions were natural logarithmically transformed before analysis. Data are presented as mean ± standard deviation (SD) for continuous variables, medians and interquartile range for skewed distributions, and counts (percentages) for categorical variables. Comparisons of the demographic characteristics, clinical features, and laboratory results between subjects in the preterm group and those in the term group were performed using Student’s *t*-test. A general linear model with adjustment for confounders was used to compare the hormone levels among the subgroups of preterm infants. Multivariate linear regression models were used to estimate the influencing factors of leptin, adiponectin, insulin, and ghrelin in cord blood after adjusting for confounders. Associations between hormones and neonatal growth adjusted for confounders were evaluated by partial correlation analysis. The confounders considered were maternal age, pre-pregnancy body mass index (BMI, in kilograms per square meter), mode of delivery, GA, and sex of the infants. A *p*-value <0.05 was considered statistically significant.

## Results

### Baseline Characteristics and Anthropometric Indices of the Mother–Infant Pairs

**Figure 1:**
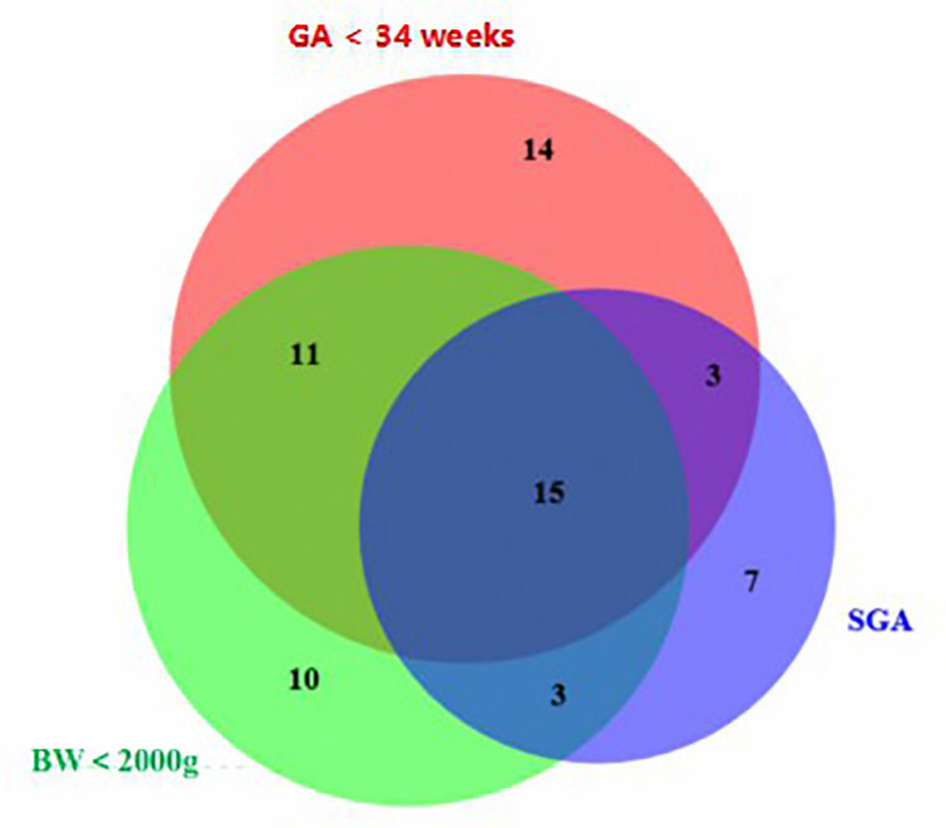
Distribution of premature infants with GA <34 weeks, BW <2,000 g, and SGA and the proportion and overlapping relationships between them. *SGA*, small for gestational age; *GA*, gestational age; *BW*, birth weight.

A total of 149 mother–infant pairs were enrolled in this study, including 100 in the preterm group and 49 in the term group. Statistically significant differences were found in GA, BW, birth length, and birth head circumference between the two groups; however, no significant differences were observed for gender, delivery mode, and maternal age (**Table 1**). The preterm group had 43 cases (43%) of GA <34 weeks and 57 (57%) of GA >34 weeks. The BW was <2,000 g in 39 cases (39%) and was ≥2,000 g in 61 cases (61%). SGA occurred in 28 cases (28%) and AGA in 72 (72%). In the preterm group, 15 cases (15%) reached the agreed standards for SGA, BW <2,000 g, and GA <34 weeks (**Figure 1**).

**Table 1:** Baseline characteristics of the mother–infant pairs in the two groups.

	Preterm group (*n* = 100)	Term group (*n* = 49)	*p*-value
Maternal age (years)	30.0 (28.0–34.0)	32.5 (30.0–36.5)	0.423
Gestational age (weeks)	34 (32–35)	39 (38–40)	<0.001***
Delivery mode (SVD/CS), *n* (%)	46 (46.0)/54 (54.0)	16 (32.7)/33 (67.3)	0.561
Gender (male/female), *n* (%)	48 (48.0)/52 (52.0)	29 (59.2)/20 (40.8)	0.252
Birth weight (kg)	2.20 ± 0.67	3.34 ± 0.4	<0.001***
Birth length (cm)	44.5 ± 3.9	49.9 ± 1.6	<0.001***
Head circumference (cm)	31.1 ± 2.4	34.3 ± 1.0	<0.001***
Adiponectin (µg/ml)	16.7 (10.6–27.4)	28.3 (23.5–36.6)	<0.001***
Leptin (ng/ml)	1.56 (0.65–3.23)	5.39 (3.99–7.99)	<0.001***
Insulin (µIU/ml)	5.80 (4.15–13.82)	5.73 (3.28–7.41)	0.865
Ghrelin (pg/ml)	374.1 (271.7–527.5)	408.4 (323.5–653.7)	0.015*

Data presented are medians, mean ± SD for continuous variables, and n (%) for categorical variables. The p-values are from t-tests for differences in means for continuous variables or chi-square tests for differences in proportions for categorical variables between the preterm and term groups. Values in bold are significant at p < 0.05.

SVD, spontaneous vaginal delivery; CS, cesarean section.

*p < 0.05, ***p < 0.001.

### Comparisons of Hormones in the Two Groups and the Subgroups of Preterm Infants

**Figure 2:**
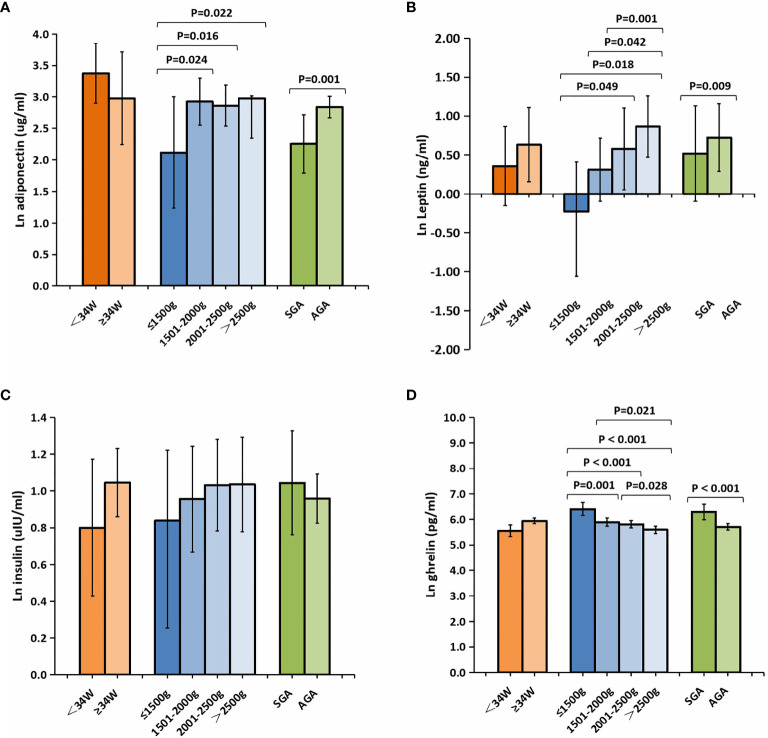
Comparison of the cord plasma concentrations of adiponectin **(A)**, leptin **(B)**, insulin **(C)**, and ghrelin **(D)** among the subgroups of preterm newborns after natural logarithmic (ln) transformation. Data were analyzed after adjusting for sex, mother’s age, delivery mode, weight gain during pregnancy, and pre-pregnancy BMI between the different GA groups, and for sex, mother’s age, delivery mode, GA, weight gain during pregnancy, and pre-pregnancy BMI among the different BW groups and the SGA/AGA groups. All data are expressed as 95% CIs. *Values in bold* are significant at *p* < 0.05. *BMI*, body mass index; *SGA*, small for gestational age; *AGA*, appropriate for gestational age; *GA*, gestational age; *BW*, birth weight.

The cord blood concentrations of adiponectin, leptin, and ghrelin in the preterm group were significantly lower than those in the term group; however, no differences in the insulin levels were found between the two groups (**Table 1**). As depicted in **Figure 2**, analysis of the preterm subgroup indicated that the concentrations of leptin were significantly lower in preterm infants with GA <34 weeks than in those with GA ≥34 weeks (mean ln leptin = 0.63 *vs.* 0.36 ng, *p* = 0.009) after adjustments for sex, mother’s age, delivery mode, weight gain during pregnancy, and pre-pregnancy BMI (**Figure 2B**). No significant differences were found for the levels of the other hormones.

The levels of leptin, adiponectin, and insulin were substantially lower in preterm infants with very low BW (<1,500 g) relative to other preterm infants (**Figures 2A–D**), whereas those of ghrelin were significantly higher. In addition, compared with infants in the AGA group, those in the SGA group showed significantly lower levels of adiponectin (mean ln adiponectin = 2.26 *vs.* 2.84 µg/ml, *p* = 0.001) (**Figure 2A**). Meanwhile, the levels of ghrelin were higher in infants from the SGA group than in those from the AGA group (mean ln ghrelin = 6.29 *vs.* 5.71 pg/ml, *p* < 0.001) (**Figure 2D**).

### Interrelationship Among Metabolic Hormones in Preterm and Term Infants

Serum leptin was significantly correlated with insulin both in preterm infants with BW >2,000 g (*p* = 0.019) and in term infants (*p* = 0.009) after adjusting for GA, sex, mother’s age, BW, weight gain during pregnancy, and pre-pregnancy BMI (**Table 2**). In addition, leptin was only found to have a significant association with serum adiponectin (*p* = 0.049) and a borderline association with serum ghrelin in term infants (*p* = 0.061).

**Table 2:** Interrelationship between the metabolic hormones in preterm and term infants.

	Adiponectin		Leptin		Insulin	
	*r*	*p*-value	*r*	*p*-value	*r*	*p*-value
Preterm infants with BW <2,000 g						
Adiponectin						
Leptin	0.223	0.318				
Insulin	−0.079	0.728	0.227	0.310		
Ghrelin	−0.179	0.426	0.204	0.363	−0302	0.172
Preterm infants with BW ≥2,000 g						
Adiponectin						
Leptin	0.144	0.351				
Insulin	0.122	0.430	0.352	0.019*		
Ghrelin	0.269	0.077	−0.060	0.701	0.060	0.697
Term infants						
Adiponectin						
Leptin	0.295	0.049*				
Insulin	0.136	0.350	0.383	0.009**		
Ghrelin	0.178	0.241	0.281	0.061	−0.041	0.792

Partial correlation analysis was used with adjustments for maternal age, parity, mode of delivery, gestational age, gender, pre-pregnancy BMI, and weight gain during pregnancy. P-values in bold are significant at p < 0.05.

r, partial correlation coefficient; BMI, body mass index.

*p < 0.05, **p < 0.01.

### Multivariate Linear Regression Analysis of the Influencing Factors for Different Hormones

GA, sex, and SGA were associated with the levels of adiponectin in umbilical cord blood. GA (*β* = 0.161, *p* = 0.001) and being female (*β* = 0.378, *p* = 0.023) were positively correlated and SGA negatively correlated (*β* = −0.642, *p* = 0.002) with the levels of adiponectin. GA (*β* = 0.248, *p* < 0.001) was correlated with higher levels of leptin. A significant correlation was found between vaginal delivery and the levels of insulin (*β* = 0.622, *p* = 0.041), while GA was negatively correlated (*β* = −0.057, *p* = 0.007) and SGA positively correlated (*β* = 0.362, *p* < 0.001) with the concentrations of ghrelin (**Table 3**).

**Table 3:** Multivariate linear regression analysis of the influencing factors for the levels adiponectin, leptin, true insulin, and ghrelin in cord blood.

	*β*	*p*-value	95% CI	
ln Adiponectin				
Maternal age	−0.015	0.444	−0.053	0.023
Parity	0.104	0.667	−0.376	0.584
SVD (yes = 1)	−0.204	0.265	−0.566	0.158
Gestational age	0.161	0.001**	0.066	0.256
Gender (female = 1)	0.378	0.023*	0.055	0.702
Pre-pregnancy BMI (kg/m^2^)	0.001	0.937	−0.016	0.018
Weight gain during pregnancy	0.001	0.937	−0.016	0.018
SGA (yes = 1)	−0.642	0.002**	−1.049	−0.235
ln Leptin				
Maternal age	−0.058	0.029	−0.111	−0.006
Parity	0.515	0.124	−0.145	1.175
SVD (yes = 1)	0.083	0.741	−0.416	0.581
Gestational age	0.248	<0.001***	0.117	0.379
Gender (female = 1)	0.307	0.174	−0.138	0.752
Pre-pregnancy BMI (kg/m^2^)	0.012	0.318	−0.012	0.035
Weight gain during pregnancy	0.012	0.318	−0.012	0.035
SGA (yes = 1)	−0.141	0.617	−0.701	0.419
ln Insulin				
Maternal age	0.013	0.711	−0.056	0.081
Parity	−0.223	0.608	−1.087	0.641
SVD (yes = 1)	0.622	0.041*	0.031	1.274
Gestational age	−0.033	0.701	−0.205	0.139
Gender (female = 1)	0.012	0.967	−0.571	0.595
Pre-pregnancy BMI (kg/m^2^)	0.021	0.18	−0.01	0.052
Weight gain during pregnancy	0.021	0.18	−0.01	0.052
SGA (yes = 1)	0.216	0.559	−0.517	0.948
ln Ghrelin				
Maternal age	0	0.961	−0.017	0.016
Parity	0.013	0.9	−0.194	0.22
SVD (yes = 1)	−0.135	0.088	−0.291	0.021
Gestational age	−0.057	0.007**	−0.098	−0.016
Gender (female = 1)	0.016	0.818	−0.123	0.155
Pre-pregnancy BMI (kg/m^2^)	−0.003	0.454	−0.01	0.005
Weight gain during pregnancy	0	0.95	−0.014	0.015
SGA (yes = 1)	0.362	<0.001***	0.187	0.538

Multivariate linear regression analysis was used with adjustments for maternal age, parity, mode of delivery, gestational age, sex, SGA, pre-pregnancy BMI, and weight gain during pregnancy. Values in bold are significant at p < 0.05.

SVD, spontaneous vaginal delivery; BMI, body mass index; SGA, small for gestational age; ln, natural logarithmic.

*p < 0.05, **p < 0.01, ***p < 0.001.

### Partial Correlations Between Different Hormones and Neonatal Growth

The partial correlation coefficients between the different hormones and the neonatal growth parameters are shown in **Table 4**. After adjusting for GA, sex, mother’s age, delivery mode, and pre-pregnancy BMI, the levels of cord blood leptin were found to be positively correlated with BW (*p* < 0.001), body length (*p* = 0.012), and head circumference (*p* = 0.012) in premature infants, whereas the levels of ghrelin were negatively correlated with these parameters (BW, *p* = 0.002; body length, *p* = 0.001; head circumference, *p* = 0.002). However, no statistically significant associations were detected between the levels of adiponectin or insulin and BW, body length, or head circumference.

**Table 4:** Relationship between the levels of adiponectin, leptin, insulin, and ghrelin and the neonatal growth parameters.

	ln Adiponectin		ln Leptin		ln Insulin		ln Ghrelin	
	*r*	*p*-value	*r*	*p*-value	*r*	*p*-value	*r*	*p*-value
Birth weight (g)	−0.052	0.678	0.460	<0.001***	0.143	0.286	−0.372	0.002**
Birth length (cm)	0.106	0.403	0.311	0.012*	0.183	0.143	−0.415	0.001**
Head circumference (cm)	0.132	0.295	0.310	0.012*	0.131	0.298	−0.373	0.002**

Partial correlation analysis was used with adjustments for maternal age, parity, mode of delivery, gestational age, gender, pre-pregnancy BMI, and weight gain during pregnancy. P-values in bold are significant at p < 0.05.

r, partial correlation coefficient; BMI, body mass index.

*p < 0.05, **p < 0.01, ***p < 0.001.

## Discussion

### Main Findings

In this cross-sectional sample of preterm infants, we found that the levels of cord blood leptin were positively correlated with the neonatal growth parameters such as BW, body length, and head circumference, whereas the levels of ghrelin were negatively correlated with these parameters. Our study is the first to demonstrate the association between the levels of cord blood adiponectin, leptin, insulin, and ghrelin and the birth outcomes in premature Chinese infants. When compared with those of other preterm infants, the levels of leptin, adiponectin, and insulin were substantially lower in those with very low BW, whereas the levels of ghrelin were significantly higher.

### Data Interpretation and Comparisons With Previous Findings

Adiponectin is secreted only by fully differentiated adipocytes and is related to insulin resistance (23). In our study, we found that the concentrations of cord blood adiponectin were significantly lower in preterm infants than those in full-term newborns, which is consistent with the results of previous studies (24, 25). Terrazzan et al. (26) reported that the concentrations of cord blood adiponectin were lower in preterm infants with BW <1,500 g than those in full-term infants in Brazil. Here, we also found that the concentrations of cord blood adiponectin in preterm infants with very low BW were lower than those in other premature infants. We further observed that, in the preterm group, SGA infants had markedly lower concentrations of cord blood adiponectin compared with AGA infants. The negative influence of SGA on serum adiponectin levels is consistent with previous reports (27, 28). GA was positively correlated with adiponectin, which suggests that the quality of early postnatal growth differed between preterm and term infants. We found a significant difference in the adiponectin levels between female and male preterm neonates. Previous studies have indicated that a higher level of adiponectin in female cord blood compared with that of males could be another indication of the advantages females have regarding the acquisition and functionality of adipose tissue during gestation (25, 29).

Leptin is an anorexigenic peptide secreted by adipocytes that enables communication between lipid tissue and the hypothalamus. Studies have shown that preterm infants have lower levels of leptin than do term infants (1), which is consistent with our findings. GA is the most commonly reported cord blood leptin modulator (30, 31); we also observed that GA was positively correlated with the levels of leptin in the umbilical cord blood of preterm infants. A positive correlation was detected between leptin and BW, birth length, and head circumference, an association that had already been suggested in a previous study in China (32). The level of leptin in umbilical cord blood increases with advancing pregnancy (33). A marked increase in the concentrations of circulating leptin after 34 weeks of gestation may be of physiological advantage to newborn infants through limiting the body energy expenditure and conserving nutritional reserves for growth and development (12). It has been suggested that the lower the leptin level, the higher the BMI and the faster the weight growth of premature infants, which is expected to contribute to the catch-up growth of premature infants.

Ghrelin is a strong orexigenic peptide hormone produced primarily in the gastrointestinal tract and, to a lesser extent, in the placenta and other tissues (12). In our study, cord blood ghrelin was detectable and its concentrations were significantly lower in preterm infants than those in term infants, which is inconsistent with previously reported results (34, 35). The association between ghrelin and GA in preterm infants is controversial (13, 25, 35, 36). Here, we found that ghrelin was inversely related to GA in premature infants. The different sampling times for ghrelin and racial differences may have contributed to the discrepant results. SGA preterm infants had higher levels of ghrelin than did their AGA counterparts. Although several studies have demonstrated an association between GA and the level of ghrelin in term infants, evidence for such an association in preterm infants has been limited (37–40), and whether a relationship exists between ghrelin and the anthropometric parameters at birth remains controversial (39, 41, 42). The inverse relationship between ghrelin and the anthropometric indices in preterm infants found in this study suggests that ghrelin might adopt its physiological role in regulating growth and metabolism at a relatively early stage of gestation.

The pancreatic hormone insulin plays a role in fetal growth (14). In this study, we found no significant differences in cord blood insulin between preterm and term infants, which does not agree with the findings of a previous study (34). The levels of cord blood insulin were observed to be higher in infants born through vaginal delivery than in those born through cesarean section, and the reason may be that cesarean section and spontaneous vaginal delivery induce different levels of stress. In our study, we did not find a correlation between the insulin levels and the fetal anthropological measurements in preterm infants; however, a positive correlation was identified between the levels of leptin and insulin and GA, BW, height, and head circumference (34).

Because hormone-mediated cross-talk between endocrine organs is thought to contribute to fetal development, we also investigated the interrelationship among these metabolic hormones in our cohort. We identified a significant correlation between cord blood leptin and insulin in both preterm infants with BW >2,000 g and in term infants, supporting that the “adipoinsular axis” is likely to be active in early intrauterine life (43, 44). The strong correlation detected between these hormones suggests that the activity and the function of the “adipoinsular axis” are likely to increase as fetal maturation progresses.

### Strengths and Limitations

One of the strengths of this research was that we not only compared the levels of adiponectin, leptin, insulin, and ghrelin in the umbilical cord blood between premature and full-term infants but also divided premature infants into subgroups according to GA, BW, and AGA/SGA. We further investigated the interrelationship among these metabolic hormones in preterm and term infants and explored whether a functional “adipoinsular axis” might exist in preterm newborns. However, there are also some limitations to our study. Firstly, we did not observe a link between ghrelin and the other hormones in either preterm or term infants, which might be due to the relatively small sample size. Secondly, given that adiponectin multimers rather than total adiponectin have been reported to be more strongly related to fetal growth and neonatal body composition (45), we only measured the levels of total adiponectin and not its multimeric forms, which might have contributed to the lack of association between adiponectin and the neonatal growth parameters in our study.

### Conclusion

In summary, our results demonstrate that hormone dysregulation occurs in preterm infants, especially those with a BW ≤1,500 g. GA and BW were found to be major determinants of the cord blood adipokine profile and the levels of ghrelin. Furthermore, the interrelationship between leptin and insulin found in term infants and in preterm infants with BW >2,000 g in this study suggests that a fetal adipoinsular axis at birth may influence the “programming” of satiety and body metabolism, thereby determining postnatal weight gain and adiposity. Combined, these findings indicate that the lack of proper acquisition of adipose tissue by the fetus in prematurity is associated with changes in the cord blood adipokine profile and the levels of ghrelin, which may disturb the metabolic adaptation of the infant to extrauterine life. These observations further suggest that the detection of the levels of cord blood hormones can reflect the development of preterm infants. However, the mechanisms underlying the observed associations need further investigation.

## Data Availability Statement

The original contributions presented in the study are included in the article/supplementary material. Further inquiries can be directed to the corresponding authors.

## Ethics Statement

The studies involving human participants were reviewed and approved by the Ethics Committee of Peking Union Medical College Hospital. Written informed consent to participate in this study was provided by the participants’ legal guardian/next of kin.

## Author Contributions

ML and DW conceived the study. LH and ZL contributed significantly to manuscript preparation. LH, BL, ML, XX, and SL performed the data analysis and wrote the manuscript. ML and DW helped with data analysis. All the authors contributed to the manuscript and approved the submitted version.

## Funding

This work was partly supported by a grant from Capital’s Funds for Health Improvement and Research (2020-2Z-40117).

## Conflict of Interest

The authors declare that the research was conducted in the absence of any commercial or financial relationships that could be construed as a potential conflict of interest.

## Publisher’s Note

All claims expressed in this article are solely those of the authors and do not necessarily represent those of their affiliated organizations, or those of the publisher, the editors and the reviewers. Any product that may be evaluated in this article, or claim that may be made by its manufacturer, is not guaranteed or endorsed by the publisher.
